# EEG Dynamics Reflect the Distinct Cognitive Process of Optic Problem Solving

**DOI:** 10.1371/journal.pone.0040731

**Published:** 2012-07-16

**Authors:** Hsiao-Ching She, Tzyy-Ping Jung, Wen-Chi Chou, Li-Yu Huang, Chia-Yu Wang, Guan-Yu Lin

**Affiliations:** 1 Institute of Education, National Chiao-Tung University, Hsinchu City, Taiwan, Republic of China; 2 Institute for Neural Computation, University of California San Diego, San Diego, California, United States of America; 3 Adult and Continuing Education, National Chung-Cheng University, Chiayi County, Taiwan, Republic of China; Tel Aviv University, Israel

## Abstract

This study explores the changes in electroencephalographic (EEG) activity associated with the performance of solving an optics maze problem. College students (N = 37) were instructed to construct three solutions to the optical maze in a Web-based learning environment, which required some knowledge of physics. The subjects put forth their best effort to minimize the number of convexes and mirrors needed to guide the image of an object from the entrance to the exit of the maze. This study examines EEG changes in different frequency bands accompanying varying demands on the cognitive process of providing solutions. Results showed that the mean power of θ, α1, α2, and β1 significantly increased as the number of convexes and mirrors used by the students decreased from solution 1 to 3. Moreover, the mean power of θ and α1 significantly increased when the participants constructed their personal optimal solution (the least total number of mirrors and lens used by students) compared to their non-personal optimal solution. In conclusion, the spectral power of frontal, frontal midline and posterior theta, posterior alpha, and temporal beta increased predominantly as the task demands and task performance increased.

## Introduction

Problem solving has been widely studied over many decades. Cognitive psychologists have categorized problems according to whether or not the problems have clear paths to a solution [1]. The problems with clear paths are termed well-structured problems; those without clear paths are ill-structured problems [2]. Scientific problem solving has not been thoroughly examined, although problem solving has been considered to be a pivotal ability for students [3]–[5] and cultivating problem-solving abilities has been seen as an important goal in physics education [6]–[8] for more than two decades. Thus, the current study specifically explores the cognitive process of solving ill-structured scientific problems – specifically closed problems with open solutions – involving optics knowledge.

Electroencephalography (EEG) has been used to probe the brain activity involving problem solving [9]–[10]. Jausovec and Jausovec [11] reported an increased inter-hemispheric cooperation in the upper alpha band for solving problems with higher levels of creativity. Kounios and Beeman [12] found that a burst of alpha-band (approximately 10 HZ) activity, followed by a gamma-band burst, was observed for problems being solved with insight. Molle et al [10] found that during spontaneous problem solving, predominantly functional types showed reduced EEG dimensionality over the anterior cortical sites, whereas predominantly predicative types showed a reduction in EEG dimensionality over the posterior parietal region [10]. Greater neural activity was also found over the temporal lobes of both cerebral hemispheres and the mid-frontal cortex before problems were solved with insight [12]. The researchers further reported that there was more neural activity measured over the posterior (visual) cortex before the presentation of problems that were to be solved analytically. These studies implied that greater EEG activities in specific frequency bands were observed in certain brain areas according to the types of problems being solved. Thus, this study aims to explore the EEG dynamics in certain brain areas while subjects solved a scientific knowledge dependent problem.

Although many studies, including those mentioned above, have tried to assess brain activity during problem solving, the problems often did not require a high level of scientific knowledge. In contrast, our study involves solving a highly scientific, knowledge-dependent, and ill-structured problem with open solutions in optics, within a Web-based learning environment. The task itself requires individuals to use optic knowledge and principles, including the image formation of convexes and mirrors, to provide at least three solutions using up to ten mirrors and three convexes. They were instructed to use as few mirrors and convexes as possible. The task involved reasoning, mental manipulation and rotation of mirrors and convexes to form an upside down image with the combination of mirrors and convexes. To the best of our knowledge, no study has systematically assessed the EEG dynamics across different frequency bands and scalp locations in problem solving. Moreover, if and how EEG activities correlate with scientific problem-solving performance is largely unknown in the literature. Thus, the relationship between EEG activity and task performance of optics problem solving were also examined in this study.

Many studies have suggested that low-frequency bands, such as theta and alpha, are associated with working memory engagement [13]–[14], and high-frequency bands such as beta and gamma serve as markers for cognitive processing, integration, and learning[15]–[16]. Gevins et al [17] reported that subjects’ frontal theta activity increased with task difficulty while performing working memory (WM) tasks. Other studies also reported that power in the theta band (4–8 Hz) increased with greater levels of mental effort or cognitive challenge [18]–[19]. Several studies also reported that neural transmission caused alpha oscillations to travel from anterior to posterior sites, suggesting that the spreading direction of alpha activity reflected some sort of communication between brain areas [20]–[21]. Sauseng et al [21] reported that upper-alpha oscillations in the frontal areas lead those in the posterior ones during top-down processing in a visuo-spatial task. Other studies reported increased alpha amplitude mainly during retention of visual or verbal material in WM [22]. The beta rhythm is accepted as a marker for cognitive processing, with integration and learning, depending on the experimental paradigm [15]–[16]. Kim et al [23] found an increase in beta power in temporal areas in a virtual-reality object-finding experiment. From the literature, we hypothesized that theta, alpha, and beta power would increase as the task demands increased, as students put forth their best effort to decrease the number of lenses and mirrors used in the solutions.

## Methods

### Participants

A total of thirty-seven (37) university students participated in this study. Of the 37 participants, 11 (29.7%) were female and 26 (70.3%) were male. Also, seven (18.9%) of them were freshmen, nine (24.4%) were sophomores, eight (21.6%) were juniors, and 13 (35.1%) were seniors. All participants majored in science and/or engineering and were recruited from two top universities in Taiwan. All participants signed a consent form regarding the process of the experiment. The Institutional Review Board of China Medical University Hospital reviewed and approved all the details regarding the experimental design. Ten of the students (27.0%) majored in physics, and the remaining 27 (73.0%) majored in non-physics fields including chemistry, mathematics, electronics engineering, and computer science. All of them had learned the required concepts for solving the optics problem described below from high school physics classes. In order to ensure their level of understanding of optic concepts, we used an eight-item Optics Conception Test (OCT, score ranged 0–8 points) to measure their prior knowledge in image formation of mirror and convex lens. The mean of the test score was 5.76±1.88.

### Experimental Task and Procedure

**Figure 1:**
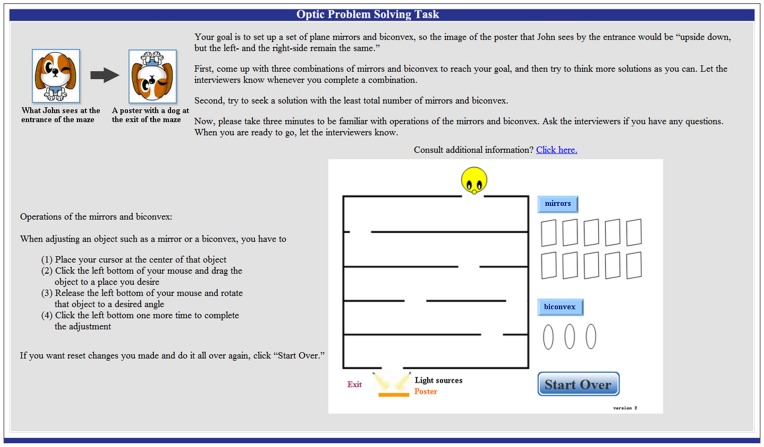
Optic problem-solving task.

The researchers developed a problem-solving task in a multimedia format, consisting of an open problem in the domain of optics (Fig. 1). In order to solve the problem, participants were required to use optics principles involving mirrors and convexes. There was no one right answer for the problem, i.e. an open solution. The problem was stated as “John wants to challenge a maze in an amusement park. He is standing at the entrance of the maze and a normal poster of a dog is posted at the exit of the maze. There are ten mirrors and three convexes available. Place mirrors or convexes along a path through the maze. With the use of convexes and mirrors, you should be able to see the poster upside down in the mirror closest to John by the entrance. With the mouse, the mirrors and convexes can be moved around and rotated to the angle of your desire. Also, you don’t have to use all of them.” Participants were asked to solve the problem on a computer without any time constraints, while their EEG data were recorded and the experiments were videotaped.

The task requires subjects to use their optics knowledge and principles, including the law of reflection and refraction, image formation of convexes, image formation of mirrors, etc. The students would need to reason whether odd or even numbers of convexes and mirrors would be used to produce an upside down image. These processes would involve reasoning and mental manipulation and rotation of mirrors and convexes to form an upside down image with the combination of mirrors and convexes, particularly when they were trying to generate an optimal solution with the least number of mirrors and convexes.

Before participants started the problem-solving task, they were given opportunities to practice manipulating mirrors and convexes with a mouse on a computer. They were asked to provide three different solutions. One of the important aims for them was to seek a personal optimal solution (the least total number of mirrors and lenses) as they progressed from the first solution to the third solution. The participants also needed to provide explanations for each solution immediately after their arrangements of mirrors and convexes in the optical maze. The EEG data during explanation period were not included in the analysis.

### Instruments

#### Optics conception test (OCT)

The Optics Conception Test (OCT) is an eight-item domain-specific knowledge test in a multiple choice format developed by researchers. The test assessed the participants’ prior knowledge of concepts central to the optics problem’s solution. Three items were related to the concepts of image formation using mirrors and five items were related to the concepts of image formation using convex lenses. Each item was worth one point. Hence, the maximum score for the pre-test was eight points. The Cronbach’s alpha is 0.701.

#### EEG recording and quantification

The EEG signals with a sampling rate of 200 Hz were recorded using a Neuron-Spectrum-5 EEG amplifier (Neurosoft Ltd., Ivanovo, Russia) from 19 electrodes (Fp1, Fp2, Fz, F3, F4, F7, F8, T3, T4, T5, T6, Cz, C3, C4, Pz, P3, P4, O1 and O2) in accordance with an international 10–20 system. All electrodes were referenced to linked mastoids (A1 and A2), and a single ground electrode was attached to participants' foreheads. Electrode impedance was maintained below 20 kΩ. All EEG data were digitally filtered with a bandpass of 0.5–35 Hz. Independent component analysis (ICA) under the EEGLAB and MATLAB® platform was used to remove artifacts from blinks or eyes. During the data preprocessing period, we set 1000 microvolts of amplitude value and five mean standard deviations as the value limitation for noise data rejection. EEG time series was converted into the frequency domain using a 512-point fast Fourier transform (FFT), resulting in a frequency resolution of 0.39 Hz. The five seconds of resting EEG data before problem solving served as the baseline section, during which we instructed subjects to relax and not think about the problems. We then subtracted the mean baseline log power spectrum from spectral estimates, producing the baseline-normalized spectra. This procedure was used to obtain resting-state normalized spectra for each solution (Solutions 1–3). The repeated measure of ANOVA was then used to examine the spectral differences among solutions in theta (θ, 4–7.9 Hz), lower-alpha (α1, 7.9–10 Hz), upper-alpha (α2, 10.1–12.9 Hz), lower-beta (β1, 13–17.9 Hz) and upper-beta (β2, 18–24.9 Hz) bands.

## Results

### EEG Activity Across Three Solutions

#### Behavioral data vs. averaged EEG power

**Figure 2:**
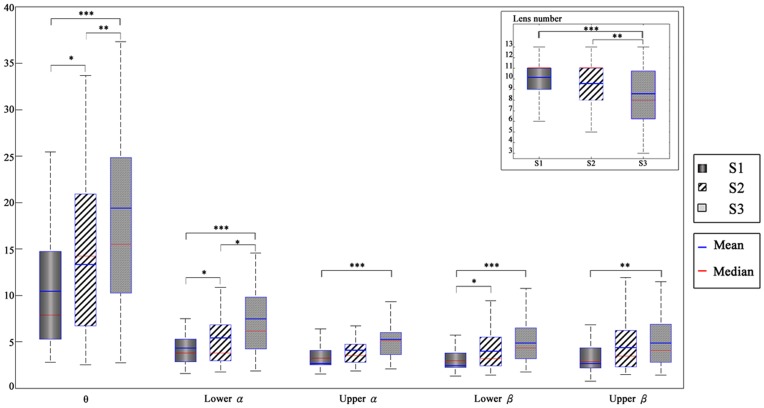
The number of lenses and mirrors used across three solutions and the corresponding EEG power in the different frequency bands.

Figure 2 shows the means and distributions of task performance and average EEG power of participants across three solutions to the optical maze. The means of number of lenses used by students tended to gradually decrease from solutions 1 (S1) to 3 (S3) (Figure 2, upper right). The repeated measures of ANOVA showed that the effect of the mean number of lenses used from solution 1 to 3 reached a statistically significant level across three solutions (Figure 2, upper right) (F = 11.71, p = 0.001). The post hoc analysis indicated that S3 was statistically significant greater than S1 (p = 0.000) and S2 (p = 0.000). The mean power of EEG activities gradually increased from S1 to S3 in all frequency bands as the number of lens used decreased. The repeated measures of ANOVA were performed to examine the effect of the three different solutions on the power of each frequency band of the EEG. Results indicated that the spectral differences were statistically significant in the θ (F = 12.75, *p* = 0.000), α1 (F = 12.93, *p* = 0.000), α2 (F = 4.34, *p* = 0.030), and β1 (F = 5.6, *p* = 0.009) power. The post hoc (Sidak test) was also used here and found that the mean power of S2 was significantly higher than that of the S1 in the θ (*p* = 0.047), α1 (*p* = 0.015), and β1 (*p* = 0.042) bands, the mean power of S3 was significantly higher than that of the S1 in the θ (*p* = 0.000), α1 (*p* = 0.000), α2 (*p* = 0.000), β1 (*p* = 0.000) and β2 (*p* = 0.007) bands, and the mean power of S3 was significantly higher than that of the solution 2 in the θ (*p* = 0.003) and α1 (*p* = 0.031) bands.

#### Topographic maps of theta power

**Figure 3:**
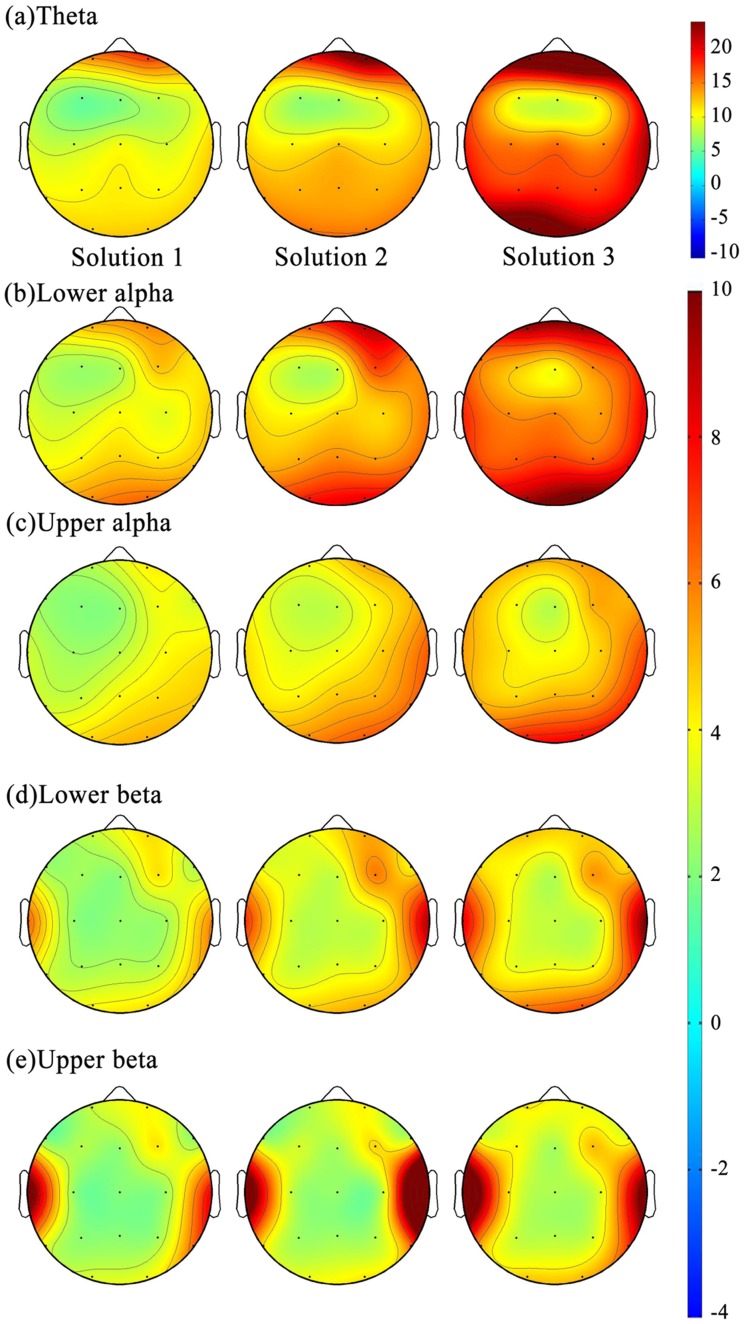
The amplitudes of EEG power at different frequency bands (rows) under different solutions.

Figure 3 shows the amplitudes of EEG power at different frequency bands (rows) under different solutions (columns). As shown, greater theta activity was observed in the frontal area (Fp1, Fp2, F7, F8) and posterior area (O1, O2) (Figure 2, top row), and the strengths of θ power in the frontal and posterior areas were fairly close to each other across three solutions. The repeated measure of ANOVA was used to compare the differences of theta power among three solutions in the frontal, posterior and frontal midline (Fz) regions. The result indicated that the mean θ power at frontal (F = 7.38, *p* = 0.003), posterior (F = 11.56, *p* = 0.001) and frontal midline regions (F = 10.82, *p* = 0.003) was statistically significantly different among the three solutions. The post hoc analysis revealed that the θ power of S3 was greater than that of the S1 and S2 at the frontal (S3>S2, *p* = 0.014; S3>S1, *p* = 0.003), posterior (S3>S2, *p* = 0.004; S3>S1, *p* = 0.001) and frontal midline regions (S3>S2, *p* = 0.001; S3>S1, *p* = 0.003). The θ power of S2 was also greater than that of the S1 at the posterior area (S2>S1, *p* = 0.020).

#### Topographic maps of alpha power

Greater lower alpha power (α1) and upper alpha power (α2) were observed in the posterior areas (O1 and O2), followed by the frontal areas (Fp1, Fp2, F7, F8, Figure 3, second and third rows) from S1 to S3. The T-test was used to examine the differences of the mean power of α1 and α2 between frontal and posterior areas across three solutions. The results indicated that the mean power of α1 and α2 of the posterior area were statistically significantly greater than that of the frontal area in S1 (T_α1_ = 2.88, *p* = 0.008; T_α2_ = 3.82, *p* = 0.001), S2 (T_α1_ = 3.42, *p* = 0.002; T_α2_ = 3.42, *p* = 0.002) and S3 (T_α1_ = 2.32, *p* = 0.028; T_α2_ = 2.27, *p* = 0.032). The repeated measure of ANOVA was then used to compare the differences of α1 and α2 power among three solutions at the posterior areas. Results of posterior α1 and α2 power were statistically significantly different among three solutions (F_α1_ = 12.22, *p* = 0.001; F_α2_ = 9.42, *p* = 0.002). The post hoc analysis revealed that α1 power of S3 was greater than that of S2 and S1 at the posterior areas (S3>S2, *p* = 0.010; S3>S1, *p* = 0.000), and S2 was greater than that of S1 (*p* = 0.003). A similar pattern was found for the posterior α2 power; that is, S3 was greater than that of S2 and S1 (S3>S2, *p* = 0.003; S3>S1, *p* = 0.003).

#### Topographic maps of beta power

Greater lower-beta (β1) and upper-beta (β2) power was observed at the temporal and frontal areas (Figure 3, lower two rows). Moreover, the mean power of β1 and β2 was statistically significantly greater at the temporal area than at the frontal area in S1 (T_β1_ = 3.73, *p* = 0.001; T_β2_ = 3.90, *p* = 0.001), S2 (T_β1_ = 3.68, *p* = 0.001; T_β2_ = 2.27, *p* = 0.031) and S3 (T_β1_ = 3.58, *p* = 0.001; T_β2_ = 4.78, *p* = 0.000). The repeated measure of ANOVA was further performed to compare temporal β1 and β2 power among the three solutions and only temporal β1 power was statistically significant different (F_β1_ = 7.90, *p* = 0.001). The post hoc indicated that S3 was greater than that of both S2 (*p*_β1_ = 0.022) and S1 (*p*_β1_ = 0.002). Although the number of lenses used by participants tended to gradually decrease from solutions 1 to 3, many subjects constructed their personal optimal solution (POS) in S2 or even in S1. Therefore, the EEG power in S3 did not refer to the subject’s best performance. To avoid confounding the problem, and to assess the relationship between EEG dynamics and task performance, the subjects' task performance and corresponding EEG power were divided into two groups – personal optimal solution vs. non-personal optimal solutions (non-POS) – for further analysis.

### EEG Activity in POS vs. Non-POS

#### Behavioral data vs. averaged EEG power

**Figure 4:**
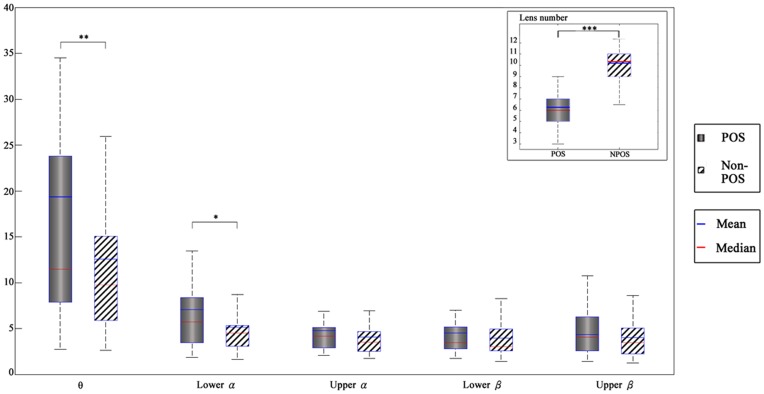
A comparison between EEG power in the different frequency bands grouped by task performance (personal optimal solutions versus other solutions).

Figure 4 shows the distributions of EEG power at different frequency bands when the subjects achieved their POS vs. non-POS. On average, the mean numbers of convexes and mirrors used in the POS and non-POS were 6.4 and 10.08, respectively (Figure 4, upper right). The difference in the number of convexes and mirrors used by subjects between POS and non-POS was statistically significant (T = 17.39, p = 0.000). In general, the mean power of EEG activity in the POS was greater than that of the non-POS across all frequency bands. Statistically significant differences in the θ (T = 3.37, *p* = 0.002) and α1 (T = 2.50, *p* = 0.017) between the POS and non-POS were found by T-test. In short, EEG power increased when the participants constructed their personal optimal solutions compared to non-personal optimal solutions.

#### Topographic maps of theta power

**Figure 5:**
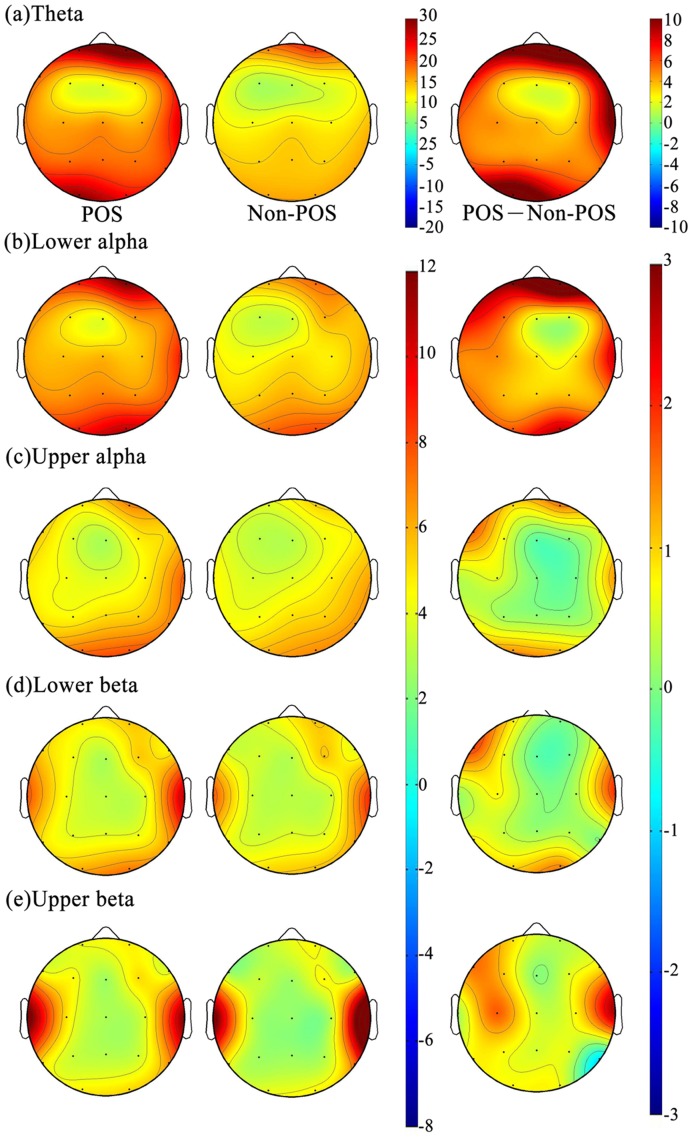
The amplitudes of EEG power in the different frequency bands under POS and non-POS conditions.

Figure 5 shows the amplitudes of EEG power at different frequency bands (rows) under POS and non-POS conditions (different columns). The greater θ power was observed at the frontal and posterior area for both POS and non-POS (Figure 5, top row), and the strength of θ power in the frontal and posterior areas fairly close to each other for both conditions. The T-test was used to compare the amplitudes of EEG θ power between POS and non-POS at frontal, posterior and frontal midline regions. The mean power of θ in the POS was greater than that of non-POS and reached statistically significant differences level, regardless of frontal (T = 2.37, *p* = 0.026), posterior (T = 2.38, *p* = 0.026) and frontal midline regions (T = 2.20, *p* = 0.037).

#### Topographic maps of alpha power

The greater α1 and α2 power were observed in the posterior areas, followed by the frontal areas, regardless of POS and non-POS conditions (Figure 5, second and third rows). The T-test was used to compare the α1 and α2 power between the frontal and posterior areas in POS and non-POS conditions. It indicated that the mean powers of α1 and α2 at the posterior area were significantly greater than that of the frontal area in POS (T_α1_ = 2.19, *p* = 0.038; T_α2_ = 2.06, *p* = 0.05) and non-POS (T_α1_ = 3.43, *p* = 0.002; T_α2_ = 3.76, *p* = 0.001). Further, the repeated measure of ANOVA was used to compare the α1 and α2 power differences between POS and non-POS at the posterior areas; however, no statistically significant differences were found between POS and non-POS conditions’ α1 and α2 power.

#### Topographic maps of beta power

The greater β1 and β2 activities were observed in the temporal areas, followed by the frontal areas (Figure 5, lower two rows). The comparison of β1 and β2 powers between the frontal and temporal areas showed that the beta power in the temporal area was significantly greater than that of the frontal area, for both POS (T_β1_ = 3.09, *p* = 0.005; T_β2_ = 4.37, *p* = 0.000), and non-POS (T_β1_ = 3.78, *p* = 0.001; T_β2_ = 2.19, *p* = 0.038) (Figure 4, lower two rows). The T-test was further performed to compare POS and non-POS solutions for both β1 and β2 power at the temporal area, and none of them reached a statistically significant level.

## Discussion

Our findings showed that greater theta activity was observed in the frontal and posterior areas across three solutions. Cohen and Miller [24] suggested that the prefrontal cortex (PFC) is responsible for cognitive control of activation and maintenance of information processing, retrieval of information from long-term memory, coordination and monitoring of working memory. The prefrontal lobe is thought to be involved in executive functions of the brain: problem solving, judgement, attention, working memory, and motor programming. Many studies have indicated that frontal theta activity is closely related to enhanced attention and that sustained neuronal activity is necessary to actively maintain the memory of representations [14], [25]–[26]. Other studies report that theta increase was observed in the occipital, parietal, and temporal lobes during a working memory task [27]. Fries et al [28] suggested that occipital theta oscillations are related to early sensory processing, such as attention or working memory. These studies provided possible explanations that the frontal and posterior theta activity might contribute to cognitive control of activation and maintenance of information processing, and early sensory processing such as attention during scientific problem solving, respectively.

Our results further demonstrated that theta power in the frontal and frontal midline regions increased as the number of lenses used in the solutions decreased. Many studies have suggested that frontal midline theta is related to mental effort, concentration and attention [14], [29]–[30], and that frontal theta activity increases with the task difficulty and memory load in WM tasks [17], [26], [31]. Miller and Cohen [32] further reported that tasks demanding greater control elicited stronger activity within the PFC. Jensen et al [25] reported that frontal theta is involved in active maintenance and recall of working memory representations. For our problem-solving task, it is a serious challenge for students to construct a solution using increasingly fewer mirrors and convexes. Students might follow a rule or a mental model which they considered to be a feasible approach in previous solution(s), but they then needed to take a step further to construct a better solution. We suspect that although the task (problem) complexity remained the same, the task difficulty increased in sessions with optimal solutions, i.e., fewer lenses, which was accompanied by increased theta power in the frontal and frontal midline regions. Our results were consistent with the above studies. It is possible that the mental effort, attention level or working-memory load increased as the number of lenses and mirrors used in the solutions decreased; thus frontal and frontal midline theta power increased from S1 to S3, and from non-POS to POS.

Greater lower alpha and upper alpha power were observed in both frontal and posterior areas. In addition, the mean powers of lower alpha and upper alpha were significant greater in the posterior area than in the frontal area across solutions. Our results support the theorem that alpha oscillations travel from anterior to posterior sites by neural transmission [21], [33]; travelling alpha may reflect a spread of cortical activation in the sense that a brain region controls, in a top-down manner activation, another region [34]. Moreover, Klimesch et al [35] reported that activity within the upper-alpha band correlated with search and retrieval processes of semantic information stored in cortical associative networks and verbal cognitive/visuospatial processing. This suggests that the alpha activities in frontal sites lead that of posterior sites during manipulation. Therefore, they suggested that anterior sites control, in a top-down manner, mental operation on the memory trace stored at posterior sites. Taking into consideration the above studies, it seems reasonable to suggest that optic problem solving might involve searching and retrieving information from semantic LTM, mental operation, visuospatial information processing, and manipulating optic image formation.

Our results showed that the beta power was significantly higher in the temporal area than in the frontal sites, throughout solutions 1, 2, and 3, POS and non-POS conditions. The results also showed that the lower and upper beta power in solution 3 was statistically significantly greater than that of solution 2 and 1. The beta rhythm has been thought of as a marker for cognitive processing, integration and learning, depending on the experimental paradigm [15]–[16]. Kim et al [23] found an increase in beta power in temporal areas in a virtual-reality object-finding experiment. Nikolaev et al [36] proposed that the increased beta oscillations indicated greater mental operation needs. The optic problem in this study required subjects to use their optics knowledge and principles to mentally manipulate images formed by mirrors and convexes to make an upside down and/or left-right reverse image. Taking into consideration the above studies, it is possible to suggest that the temporal lower-beta increased as participants employed greater mental operation and integration of information at S3 compared to S2 and S1.

### Conclusions

The study explored how EEG dynamics are associated with subjects’ task performance while solving a scientific problem. Specifically, EEG changes in different frequency bands accompanying varying demands on the cognitive process of providing solutions were examined in this study. Results showed that the EEG power of θ, α1, α2, and β1 frequency bands are negatively associated with their task performance (number of lens used in each solution) from solutions 1 to 3. Furthermore, greater theta and lower-alpha power was observed when the participants constructed their personal optimal solution (the least total number of mirrors and lens used by students) compared to non-personal optimal solution. The spectral power of fontal, frontal midline and posterior theta, posterior alpha, and temporal beta were observed predominately during the period in which the subjects generated solutions to the scientific problem, respectively. In summary, the EEG power increased as their task demands and task performance increased.
